# Patient experiences of receiving arthroscopic surgery or personalised hip therapy for femoroacetabular impingement in the context of the UK fashion study: a qualitative study

**DOI:** 10.1186/s13063-021-05151-6

**Published:** 2021-03-16

**Authors:** A. X. Realpe, N. E. Foster, E. J. Dickenson, M. Jepson, D. R. Griffin, J. L. Donovan, on behalf of the UK FASHIoN study group, Rachel Hobson, Peter Wall, Stavros Petrou, Nick Parsons, Matthew Costa

**Affiliations:** 1 Population Health Sciences, University of Bristol, Canynge Hall 4.07, 39 Whatley Road, Bristol, BS8 2PS UK; 2 Arthritis Research UK Primary Care Centre, Research Institute for Primary Care and Health Sciences, Keele University, Keele, UK; 3 University of Warwick, Coventry, UK; 4 University Hospitals of Coventry and Warwickshire NHS Trust, Coventry, UK

**Keywords:** Femoroacetabular impingement, Hip arthroscopy, Hip physiotherapy, Orthopaedic patient experiences, Qualitative study

## Abstract

**Background:**

UK FASHIoN was a multicentre randomised controlled trial comparing hip arthroscopic surgery (HA) with personalised hip therapy (PHT, physiotherapist-led conservative care), for patients with hip pain attributed to femoroacetabular impingement (FAI) syndrome. Our aim was to describe the treatment and trial participation experiences of patients, to contextualise the trial results and offer further information to assist treatment decision-making in FAI.

**Methods:**

We conducted in-depth semi-structured telephone interviews with a purposive sample of trial participants from each of the trial arms. They were interviewed after they received treatment and completed their first year of trial participation. Thematic analysis and constant comparison analytical approaches were used to identify themes of patient treatment experiences during the trial.

**Results:**

Forty trial participants were interviewed in this qualitative study. Their baseline characteristics were similar to those in the main trial sample. On average, their hip-related quality of life (iHOT-33 scores) at 12 months follow-up were lower than average for all trial participants, indicating poorer hip-related quality of life as a consequence of theoretical sampling. Patient experiences occurred in five patient groups: those who felt their symptoms improved with hip arthroscopy, or with personal hip therapy, patients who felt their hip symptoms did not change with PHT but did not want HA, patients who decided to change from PHT to HA and a group who experienced serious complications after HA. Interviewees mostly described a trouble-free, enriching and altruistic trial participation experience, although most participants expected more clinical follow-up at the end of the trial.

**Conclusion:**

Both HA and PHT were experienced as beneficial by participants in the trial. Treatment success appeared to depend partly on patients’ prior own expectations as well as their outcomes, and future research is needed to explore this further. Findings from this study can be combined with the primary results to inform future FAI patients.

**Trial registration:**

Arthroscopic surgery for hip impingement versus best conventional care (ISRCTN64081839). 28/02/2014.

**Supplementary Information:**

The online version contains supplementary material available at 10.1186/s13063-021-05151-6.

## Background

Femoroacetabular impingement (FAI) syndrome is a painful hip disorder characterised by premature contact between the proximal femur and the acetabulum (socket) [1, 2]. This painful impingement occurs as a result of certain hip shapes that increase the likelihood of premature contact. Over the last 15 years, arthroscopic hip surgery has been increasingly used to treat the FAI syndrome, whilst other patients have been treated with physiotherapy [3, 4]. Hip arthroscopy (HA) aims to reshape the hip joint to prevent the premature contact between the joint surfaces. Physiotherapist-led rehabilitation aims to improve hip muscle control and stability preventing the joint impingement [4–6].

UK FASHIoN was a pragmatic, multicentre randomised controlled trial (RCT), conducted at 23 UK National Health Service (NHS) hospitals. The trial was designed to compare two treatments. One arm of the trial was hip arthroscopy surgery (HA) which aimed to modify the anatomic structure of the hip to avoid premature contact. The other treatment arm was a course of a physiotherapist-led structured programme comprising education, activity modification, pain relief and exercise, which changed muscle co-ordination and hip movement patterns, in the FASHIoN trial referred to as Personalised Hip Therapy (PHT). 344 trial participants were randomly allocated to one of these two treatments. They completed hip-related quality of life questionnaires. The primary outcome was hip-related quality of life measured using the self-reported iHOT-33 questionnaire [7] at 12 months following randomisation. The results indicated that, on average, both treatments improved hip-related quality of life, although HA led to greater clinically significant improvement than PHT at 12 months [5].

We asked a group of trial participants what it was like to receive these treatments in the context of this trial. There is emerging recognition that patient experience is an important indicator of quality alongside information on clinical effectiveness and patient safety in orthopaedics [8, 9]. Qualitative research methods have been used to systematically explore patient experiences and the meaning attributed to them [10]. This type of research can also help with dissemination of evidence-based information to improve healthcare advice and treatment decision-making [11, 12].

The aims of this study were to describe the treatment and trial participation experiences of participants in the UK FASHIoN trial, contextualise the trial results and offer further information to assist treatment decision-making in FAI. The interviews were pre-planned as part of the trial qualitative assessment of outcomes stated in the FASHIoN trial protocol [13].

## Methods

We adopted a qualitative methods approach to focus on participants’ experience of treatments in the FASHIoN trial. Our approach was theoretically informed by critical realism applied to social science. Critical realism proposes that through scientific enquiry, we can understand enduring features of our reality; however, knowledge is transitory and always situated within a historical, social and cultural context. Therefore, the aim of any investigation is to create a plausible description or explanatory account of the object of study [14, 15]. We also adopted a social cognitive theory perspective that invites the exploration of people’s speech and behaviour to find out how they exercise personal agency in a socially coordinated and interdependent manner [16].

In accordance with this theoretical stance, we conducted in-depth semi-structured telephone interviews with a sample of participants in each of the trial arms. To obtain a wide range of experiences, we used purposive sampling to access participants with a variety of characteristics (older, younger, male, female, active, less active) based on the baseline patient questionnaires and independently from their trial outcome at 12 months (i.e. whether they perceived their allocated treatment was effective or not). We also identified and approached trial participants that theoretically were likely to yield important information to have the greatest impact in the development of understanding [17]. These participants had expressed dissatisfaction with their allocated treatment or experienced complications. Site teams notified the trial coordinator about these cases (e.g. after a conversation with a research nurse or reported complications), who in turn provided contact details to the qualitative researcher to arrange interviews. We selected participants at regular 6-month intervals from the beginning to the end of the trial and stopped once data saturation was achieved [18] Data collection was carried out from October 2014 to June 2017.

### Procedure

Trial participants received the 12-month follow-up questionnaire and, at the same time, were sent an invitation to participate in patient exit interviews, a participant information sheet, and a consent form. They were asked to send back the signed consent form with their completed trial follow-up questionnaire. The qualitative researcher then contacted them by email or telephone to arrange an interview. A topic guide is included in Additional file 1.

Interviews were audio recorded, transcribed, and analysed using NVIVO, a qualitative research software package. Transcripts were coded line by line resulting in a large number of open codes that we proceeded to group into categories that described concepts, their properties, and dimensions. Two members of the qualitative research team independently coded 10 interviews. The results were compared and disagreements resolved through discussion. Once the coding frame was agreed, AR continued to code the rest of the interviews. At the time of our analysis, a Cochrane quantitative and qualitative synthesis to improve understanding of the complex inter-relationship between pain, psychosocial effects, physical function and exercise was published [19]. We organised the categories and subcategories emerging from our analysis into themes inspired by the work reported by Hurley et al. [19]. Finally, we created narratives to explain the range of experiences described by participants in a theory building approach [17].

## Results

### Sample

Forty trial participants were interviewed for this qualitative study. We compared the thematic coding applied to the interviews constantly and found that no new themes emerged; therefore, we were confident that we achieved data saturation in the selected sample [18]. Table 1 summarises their characteristics compared to the full sample of trial participants. Interviewees’ age and levels of physical activity at baseline did not differ from those in the main trial sample. The interview sample included more males than females in the PHT arm, and one more individual in the HA arm. Hip-related quality of life scores (iHOT-33) for the interviewees were, on average, lower than those for the full sample due to the theoretical sampling of patients reporting poor outcomes in the interview sample. Time of the interview after participants submitted their 12-month questionnaire was 7 months on average. Although this period may cause issues with recall, people tend to reflect deeper on their experiences after some time has passed [20]. The median duration of the telephone interviews was 13.50 min, and the range was between 6.18 and 33.19 min.

**Table 1:** Characteristics of FASHIoN trial participants interviewed compared to the full sample of participants

	***Interviewed trial participants***		***Full sample of trial participants***	
	*HA (n = 21)*	*PHT (n = 19)*	*HA (n = 171)*	*PHT (n = 177)*
Age in years (SD)	39.7 (9.3)	37.6 (10.2)	35.4 (9.7)	35.2 (9.4)
Sex; female to male	11:10	4:15	71:100	64:163
Physical activity (UCLA Activity Scale); mean (SD)†	4.5 (2.3)	4.2 (2.7)	4.3 (2.5)	4.4. (2.5)
Hip related quality of life (iHOT-33); mean (SD)*	45.4 (21)	37.8 (23)	58.8 (21)	49.7 (25)

*HA* hip arthroscopy, *PHT* personalised hip therapy, *UCLA* University of California Los Angeles. †UCLA Activity scale scores at baseline; ranging from 1 to 10. 1 = “no physical activity, dependent on others” to 10 = “regular participation in impact sports.” *iHOT-33* International Hip Outcome Tool, visual analogue scale, ranging from 0 to 100, with 100 representing the best possible quality-of-life score. *iHOT-33 scores at 12 months

### Findings

#### Experiencing femoroacetabular impingement and seeking treatment

Participants reported to have suffered hip pain for several years before arriving at the trial clinics. They described a gradual increase of pain that became acute after specific physical activities or movements. Other common symptoms were stiffness and clicking of the hip; together with pain, these symptoms hindered physical function impeding activities of daily living.

Interviewees described how having FAI became disruptive in their lives. For example, P36 said “I had difficulty moving around because I do physical work, I’m a painter and decorator”. Another participant commented on not being able to go swimming with their children because they were “scared that if I was in the swimming pool with the kids, I would not be able to help them” (P7). There were also consequences to their mental health, P13 said “I was feeling depressed because of constant pain and at a young age”. Overall, participants’ quality of life was diminished due to FAI.

When asked about what prompted them to seek treatment, some participants said they sought treatment after an injury or accident, but most reported seeking out treatment due to increasing and more frequent pain. Eight interviewees reported receiving misdiagnoses such “dysplasia after pregnancy, sciatica and finally osteoarthritis. It was progressively getting worse” (P14). Most interviewees reported having the FAI diagnosis after seeing numerous healthcare professionals, including physiotherapists and orthopaedic surgeons. Only two out of the 40 patients interviewed reported receiving a diagnosis and referral to a hip clinic within a year of starting seeking healthcare treatment. Once they had received a diagnosis of FAI, participants were prepared to have any treatment, including surgery.

#### Experiences of receiving treatment for FAI within the FASHIoN trial

Participants arrived at the trial clinics and were invited to participate in a randomised controlled trial that allocated them to either HA or PHT. These interviewees accepted to be part of the trial and received their treatment. We interviewed 19 patients who were allocated to PHT and 21 patients who were allocated to HA. Below, we summarise patient perceptions of each treatment arm including outcomes related to hip pain and function, associated psychosocial consequences, health beliefs and future treatment plans. Example quotes are presented in Table 2.

**Table 2:** Quotes illustrating main qualitative themes organised by allocation

**Allocated to PHT**	**Hip much improved with PHT**	**Hip remained the same after PHT**	**HA after having PHT**
**Experiences of pain** Descriptions of hip pain after allocated treatment	*I am still massively better than what I was before I had the physiotherapy (P15)*	*I do not think [hip pain]‘s worse but I think it’s not any better. I do not think it’s improved (P36)*	*The three exercises I was left to do started to give me more pain and it shifted to the lumbar region as well (P10)*
**Physical function** How treatment has impacted their hip related activities	*If I just keep doing the stuff [exercises] that I was given does help, but I’m making sure that I’m stretching a lot as well (P32)*	*They are telling me to do exercises every single day and with my job and lifestyle, sometimes you cannot do these exercises (P16)*	*I mean I can go for long walks and no trouble whatsoever and then just sometimes I might get it occasionally, or depending sometimes more often than not, some pain, you know, but it’s not usually to the degree where I have to resort to painkillers. (P28)*
**Psychosocial consequences** Statements on how treatment affected mood, self and social roles	*Being out of pain made a massive difference to my mental wellbeing (P32)* *I have not had to have any time off work, and certainly sort of with a fairly physical job would be a big disadvantage (P37)*	*I literally loved going to the gym and I find it really hard to do things now. I find it hard to get motivated as well. (P26)* *I’m currently on an apprenticeship. I struggle to do parts in that so [employers] help me with certain aspects. (P34)*	*It would have been easier to stick with [PHT] but I was just getting to the point where I was fed up with it and I wanted something done basically (P23)* *It was just stopping me from fulfilling my full potential for activities like running, and it would always be in my mind (P13)*
**Health beliefs** Thoughts on causes of FAI and how treatments work	*I have experienced less pain and I think that is partly due to effectively rotating my pelvis forward. So, what is the real cause of my pain? Not necessarily the shape of my hip but rather the fact that I have a torn labrum (P9)*	*[Clinicians] were telling me that the shape of my bones, as an example, my ball and socket joint, are the wrong shape. So to grasp the concept that doing exercises is going to change the mechanics of that, you have to be quite sceptical (P11)*	*I think one of the problems with physio from my own point of view is unless you buy into doing physio yourself, you will not get better. Nobody can make you do physio apart from yourself. (P18)*
**Treatment experiences** What receiving treatment was like	*The physiotherapist introduced me to some sort of basic exercises and movements through the first couple of sessions and then we progressed with those and built on those (…*) *I found that all quite straightforward really. (P37)*	*My appointments were not particularly long, they were quite short, normally about 20 min probably tops. I probably do not know if I got that much out of it really if I’m being brutally honest. (P24)*	*Like the physio was a bit rushed, it seemed; like you go in and then you have got 15 min, boom, boom, boom, and then they show you some exercises and then you are basically left on your own again (…)* *The initial recovery [from surgery] was quite quick and obviously the scars are quite small so it does not affect you like if you are a bit conscious of your body (P13)*
**Further treatment plans** Plans for further interventions for FAI	*I do not feel I would require additional treatment in the short to medium term (P15)*	*I think that possibly surgery is the only option that is open to me (P36)*	*What I understand there is always a chance that I could develop arthritis in my hip anyway and if that’s the case I may need further treatment, but at this point in time I’m not concerned about that (P23)*
**Allocated to HA**	**Hip much improved after HA**	**Hip felt worse after HA**	
**Experiences of pain** Descriptions of hip pain after allocated treatment	*I do not think I’m to 100% yet, but I’m a lot better than I was. I was quite glad to be honest. (P1)*	*I have good days and bad days. On a bad day I have quite a lot of pain in both hips because the other hip is now starting to have the same problems but even on a good day, I am not entirely pain free. (P5)*	
**Physical function** How treatment has impacted their hip related activities	*[surgery] has made a tremendous change to my life, being able to just do the day to day thing, things that a lot of people take for granted (P14)*	*Certain movements like getting out of the car and after long walks [hip]’s very sore, I’m not doing too much running which I find that quite sore, turning and things like that so. (P39)*	
**Psychosocial consequences** Statements on how treatment affected mood, self and social roles	*I’m a lot happier in myself because I can do more things. I can walk more, I can do everyday chores better than what I did before. (P21)* *I’d literally started volunteering at a rugby club just helping out, and I was doing 12,000 steps a day, just walking. Which I’d never been able to do that amount before. So, yes, the difference was amazing (P22)*	*All I want to do is get better and go back to doing the things I love, the things I’m passionate, especially being in the water because I love surfing and wakeboarding and I miss all that because I have not been able to do it for a year. (P27)* *[I was] expecting only about six to eight months to be back on track with the type of activities I used to do before, like yoga, but that’s not been the case. (P19)*	
**Health beliefs** Thoughts on causes of FAI and how treatments work	*I do not feel that the physio would have helped. I’ve done lots of other physio and so on for various other injuries and I just felt that in my particular case, I knew my own body quite well, so it was a case of I do not know what physio could have done to help me with the pain I was getting in the coccyx. (P8)*	*What I was hoping it would repair the damage and I could, if I get back to what it used to be but at the minute it’s not. I’m not sure that it’s going to get any better. (P39)*	
**Treatment experiences** What receiving treatment was like	*The surgery was really good. I was five weeks on crutches and it was a good recovery. [The hip] just gets really tight still, that’s the only thing I’ve got now. It’s sometimes a little bit painful if I do too much but it’s a lot better than before surgery. (P25)*	*My boyfriend had to do a lot for me. I felt very debilitated. But, you know, I get that, that was just the healing process. I was only two crutches for four weeks and then one crutch for a couple of weeks. (P12)*	
**Further treatment plans** Plans for further interventions for FAI	*I do have a slight impingement in my right hip as well, but it wasn’t as bad as my left. I think it was just going to be managed by physiotherapy, if it was bothering me, but it is not to the extent that I am kind of thinking [of surgery] yet. (P14)*	*At the minute yes [planning for further intervention] because I am still doing rehab so I will have to see what the physio says. (P39)*	

*HA* hip arthroscopy, *PHT* personalised hip therapy, *FAI* Fermoroacetabular impingement, *P#* Participant unique ID number

##### Allocated to personalised hip therapy (PHT)

Of the 19 participants who were allocated to PHT, five people reported their hip pain reduced after receiving PHT. Patients whose hip improved with PHT said they managed pain much better by doing the exercises they had learned. Most had gone back to their valued physical activities but acknowledged they had to implement modifications to the type and intensity of these activities. This was based on their knowledge about what makes their hip more or less painful. Interviewees also reported that PHT helped them improve their core strength. In relation to psychosocial consequences, participants appreciated not having to take lot of time off from work and other leisure activities to recover from surgery. Being able to engage with physical activities contributed to a better quality of life for these participants.

Participants in this group said PHT was delivered by competent physiotherapists, who supported them in progressive plans designed for their individual needs. In relation to their health beliefs, PHT represented a *gentler* and less invasive approach that they did not perceive to have high health risks compared to surgery. They reported that the success of their PHT depended on their own commitment to self-care, and two participants expressed ambivalence about recovery given that PHT had not addressed the mechanical pathology in their hip that they believed FAI to represent. This group of interviewees were not seeking further treatment for their FAI, but as a group, they did express they would be willing to consider surgery if their hip pain returned or worsened.

Eight of 19 participants who received PHT reported minimum improvement or no effect on their hip pain. One person said they stopped the exercises from the PHT programme after 3 months because the pain was worse. These participants found the exercises helped them to strength their muscles and improve the mobility of the hip. However, the effects were short-lived, and they struggled to maintain an exercise routine. Only two participants were able to go back to their valued physical activities with modifications. Like participants who felt their hip improved after PHT, this group thought not having to take time off work was an advantage of the PHT treatment. They did however express annoyance that their hip pain persisted and hindered their ability to participate in physical activities.

The group who felt their hip remained the same after PHT described fewer and shorter physiotherapy sessions than they expected and exercises that did not help decrease their hip pain. Two out of eight commented on their PHT treatment being designed to meet their individual needs, but others felt the physiotherapy sessions were not frequent enough and the PHT relied on them doing their exercises on their own. Although participants considered PHT a less invasive approach than surgery, six believed that the HA surgery was likely to be a more effective treatment; the other two expressed uncertainty. Four people mentioned concerns about the high risks of surgical intervention. Overall, their expectations of being pain-free and able to engage in their valued physical activities at the end of the treatment were not met. At least four of the eight in this group explained they would look to have surgery in the future if their symptoms increased.

Finally, six patients of the 19 PHT trial participants interviewed decided to change their treatment to have HA surgery before the end or soon after the PHT treatment finished. Although three interviewees said the exercises helped ease their hip pain, they decided they needed to “do something about it”, which meant having surgery. Participants cited difficulties maintaining self-management after PHT treatment and experiencing locking, clicking, and tightening of their hip joint. These symptoms impeded their ability to engage in valued physical activities. Two people reported feeling stressed and frustrated with the lack of progress leading to their decision to proceed to surgery. The six participants, overall, reported having recovered from their HA surgery in a shorter time than they initially expected.

Most participants in the group who changed to HA described their experience of PHT as disappointing. This appeared to be related to fewer and shorter sessions, the need for continued personal adherence to their exercise programme after the PHT finished and the physiotherapist input ended, feeling the physiotherapist hurried their sessions and not seeing the improvement in their pain in the way they had hoped. They believed that PHT could not be effective because FAI was a mechanical or bone problem in the hip joint. Like the group of patients who felt their hip symptoms remained the same, their treatment expectations were not fulfilled by PHT. They emphasised that PHT depended on the person’s commitment to follow their exercise routine. This represented a challenge for people with busy lives and on-going pain. In relation to further treatment, four people explained that they would not seek any more, whilst two were considering further physiotherapy to strengthen their muscles and speed their recovery following HA surgery.

##### Allocated to hip arthroscopy surgery (HA)

Fourteen of 21 participants who received hip arthroscopy felt their hip symptoms improved after surgery. They reported a marked decreased of hip pain, although “still not hundred percent”. Most noticeable were comments about increasing range of movement in the hip joint. Interviewees described various instances of activities that were unable to do (e.g. walking long distances, sleeping throughout the night, working out) and were now performed with ease. Participants said having surgery had improved their quality of life.

Some participants reported feeling anxious about the prospect of surgery but most people mentioned being supported and well cared by the clinical team. Whilst about half of the participants perceived recovery from surgery was quick, others felt recovery took longer than expected. Most people reported it took 4–6 weeks for them to be able to put weight on their operated leg. Interviewees expressed feeling debilitated and vulnerable during this period and needed making extra arrangements to accomplish their daily activities. In relation to their health beliefs about surgery, they considered surgery was effective because it dealt with the mechanical issue of FAI. In consequence, they also thought PHT could not be as effective in their particular case. Their expectations of less hip pain were realised with this treatment. The majority of people would not seek further treatment and three people were considering surgery for their other hip.

Seven participants out of 21 who were allocated to hip arthroscopy reported having equal or worse hip pain after the surgery than prior treatment. Patients noticed certain hip movements became restricted (e.g. squats) or too painful to execute. Three participants reported increased pain in knees and back pain. This group of patients did not feel their quality of life improved with this treatment.

Similar to patients in the improved group, these participants received good care from their clinical team. However, they felt recovery took much longer than expected. They had complications after surgery including wound infections and increasing hip pain. One participant suffered a serious adverse event and was preparing for hip replacement at the time of the interview. In terms of health beliefs, this group shared similar views to the improved group, thinking surgery was the most effective treatment and had high expectations for improvement. Unfortunately, these expectations were not fulfilled. These patients considered having physiotherapy to improve their core strength and management of hip pain.

#### Experiences of participating in a randomised trial comparing treatments for FAI

We asked interviewees about their reasons to take part in a study that randomly allocated treatment for their hip condition. More than half (22 patients) thought they could contribute to science and benefit future patients. Fourteen mentioned the opportunity to access a new treatment (8 surgical and 6 PHT patients) whilst experiencing little personal burden completing questionnaires (3 surgical and 5 PHT patients). Patients also mentioned that they expected to receive more information about their condition and outcomes by participating in the study (6 surgical and 3 PHT patients). Six patients felt they received extra support from the research team (4 surgical and 2 PHT patients). Example quotes are presented in Table 3.

**Table 3:** Quotes illustrating main themes in relation to trial participation experiences

Themes	Examples
Believing participation is for the greater good	*I like to help. I’ve spent my entire adult life in public service so I think if my experiences can help somebody else, then I’m quite happy to help. The questionnaires were forthright, I did not have any problems with it. (P18)*
Accessing a novel treatment	*I found it interesting and I’m always up for anything that can be dealt with rather than surgery, so I was open minded with it. (P24)*
Experiencing little personal burden	*it’s not like a massive burden to you, it’s not going to take up a load of your time and obviously the more people that help out in these studies, obviously the better the treatments become. (P1)*
Wanting more information about their condition	*I do not honestly know [whether to seek further treatment]. That’s one thing I was hoping to find out really after the trial period is finished. To find out if I need to keep up with the exercises for another period of time. (P37)*
Receiving extra support from the research team	*I enjoyed it. Like I said it was nice being able to talk to someone [a researcher] and it did not feel like I was just turning up and we have got to do this, this and this and go home. (P32)*

However, nine patients (4 surgical and 5 PHT) felt that they could not recommend participating in trials like FASHIoN to others mainly because they did not get any benefit from their allocated treatment. For example, one patient said:

I’m sure you guys [research team] will have the answer to what’s the best way to cure hip impingement, but for me it hasn’t worked so I probably wouldn’t recommend [participating in a trial like FASHIoN] (P38)

We also found that 13 patients (6 surgical and 7 PHT) were disappointed by the lack of support from the research team once they completed their study participation. These patients were not sure if the research team or clinicians should be contacting them to make a final evaluation of their progress and explore further treatment if necessary. These two quotes from patients who received surgery and PHT respectively illustrate these feelings:

I feel a bit like I’ve just been neglected, if that makes sense, whereas... And I’m not sure which arm [was better]. Like me personally, I feel like I’m a bit in limbo (P13)

I was confused about because I know the lady at the physio said that I will get a letter from the doctor and then see him again, but since then I haven’t actually heard anything from anyone (P35)

It is important to recognise that the interviews took place before the publication of the trial results. All participants were contacted with a summary of results, a copy of the main results paper and a thank you card that could have helped participants have a sense of closure to their participation.

In relation to the questionnaires, patients said they were easy to fill out (*n* = 10) and that they helped them gain better understanding of their progress recovering from FAI (*n* = 7). Four patients expressed concerns about the accuracy of their responses, for example, one patient mentioned having to answer questionnaires after receiving a steroid injection, thus not reflecting their usual levels of pain. Three patients suggested alternative online questionnaires that were more convenient than having to post paper questionnaires back to the research team.

After reflecting on their study participation, twelve patients (5 surgical and 7 PHT) believed that future patients should be offered personalised hip therapy before considering hip arthroscopy for FAI. For example, this patient who did not experience benefits of hip surgery said:

In hindsight, knowing now what I do that didn’t seem to make much difference, I probably would have chosen the try physio first of all to see if there was any difference, but I wasn’t given an option so I was just allocated surgery (P12)

Another patient who ended having surgery after PHT remarked on how physiotherapy for the condition may help patients develop core strength that would be beneficial in recovering from hip surgery:

[Physiotherapy] is a good idea because if you've got a minor hip impingement, strengthening the muscles around the joint may well – it made mine better – so it may work, but what I do think probably has helped was because I was doing my physio right up until I had the operation, I recovered quicker (P18)

However, patients who felt that hip arthroscopy made their hip pain much better did not agree with trying physiotherapy first before accessing surgery. The following quote exemplifies this belief:

[Physiotherapy] could not work so I was glad to get the operation to be honest. With the physio [therapy] it could be like a year down the line where you have still got this pain in your hip and that extra bit of bone needs shaving off then it seems you've wasted a year recovery (P1)

## Discussion

Our qualitative findings provided context to the results of the FASHIoN trial. Participants reported improvement in their hip symptoms in both groups, allocated to HA and PHT. However, more patients (*n* = 14) who were allocated to HA felt better and that their symptoms improved compared with patients allocated to PHT (*n* = 5). This is consistent with the main outcomes of the UK FASHIoN trial that reported that HA was more clinically effective than PHT. Whilst PHT may not have been as successful with as many patients as surgery, it offered some benefits. PHT may be helpful for people who do not need or want surgery. And as some participants suggested, PHT could be offered as a first line of treatment for patients reluctant to go straight to surgery.

Limitations of this study include the brevity of the interviews, which did not allow for exploring psychosocial consequences of treatment in depth. There was also a period of time between treatment and interview that may have reduced the ability of participants to recollect important details of their experience. However, this was a relatively large sample of qualitative interviews that covered a wide range of experiences. Theoretical sampling captured the nuances of dissatisfaction and other experiences providing a rich understanding and interpretation of the trial outcomes. Our approach focused on salient aspects of patient treatment experiences. The timing of the interviews allowed time for reflection on outcomes. Another limitation was using a framework to organise key themes based on the work of Hurley et al. [19] that synthesised research papers on treatments for osteoarthritis. This is a chronic pain condition that affects older adults and differs from the population affected by FAI. However, we suggest the chosen framework captured key aspects of the experience of these orthopaedic patients.

This study contributes to understanding how preoperative expectations may affect the results of surgical and not surgical interventions for FAI. Participants in this study expected to restore their quality of life and regain wellbeing following treatment. They cared most about regaining wellbeing often represented by returning to leisure physical activities. This is often the benchmark for orthopaedic patients who submit to surgery or other invasive procedures [21]. Regardless of outcomes, patients receiving PHT expected more time with the physiotherapist and found that PHT required more of their own input than they anticipated in spending time completing their exercises at home. Patients who received HA commented that the recovery took longer than they had anticipated. The perception that FAI syndrome was a hip mechanical issue guided most patient judgements in relation to whether a treatment was permanent or not and in some cases whether it was effective in the long term. This is consistent with research findings on patient experiences that suggest a strong link between patient beliefs about pain, their attitudes and behaviours to engage with specific treatments and their appraisals of their treatment effectiveness [19, 22].

This study reported on treatment experiences ranging from satisfaction to lack of improvement. These finding alongside the trial quantitative results, which show improvements from both treatments although superior in the HA group, will enable patients to make informed choices about treatment for FAI syndrome.

In relation to trial participation, the orthopaedic scientific community has been increasingly prioritising the acquisition of high-quality evidence through RCTs [23]. In the UK, this has led to the completion and ongoing conduct of various orthopaedic trials. Consequently, an increasing number of orthopaedic patients are participating in clinical trials, providing a unique opportunity to learn about their experiences as participants in this type of research [24]. Indeed, it has been suggested that trial participants are likely to be motivated by ‘conditional altruism’ [25], where being in a trial is perceived to be good not only for society but also the individual, who may have received some personal benefit and no significant disadvantage. Our findings supported the hypothesis that patients can be motivated by conditional altruism. The perceived lack of burden in completing questionnaires, straightforward procedures and helpful research teams meant trial participants felt good about taking part and did not feel extra personal cost.

Research into trial participation suggests patients’ decisions to continue or withdraw from studies relate to their expectations of trial requirements and treatment preferences for the arms in the study [12, 23]. It was interesting that many patients expressed dissatisfaction with the follow-up procedures once their participation in the active part of the trial finished. It is possible these patients had unrealistic expectations of trial requirements which endangered retention and future participation. An explanation that resonates with the work of Woolhead et al. [26] that showed that post-facto expectations are confounded by outcomes (i.e. people with bad outcomes inevitably feel they expected better). This is an area that continues to present challenges to researchers and more information is needed [27].

## Conclusion

Future patients diagnosed with FAI and their clinicians have high-quality scientific evidence that there are at least two effective treatments: hip arthroscopy and personalised hip therapy. Our qualitative analysis of the outcomes of the UK FASHIoN demonstrated that patients’ experiences depended partly on their expectations as well as their outcomes. Information materials to facilitate patient informed choice based on the results of the main trial and the outcome nuances of this study will be required. Theoretical sampling is recommended when conducting qualitative research in patient experience in trials to capture rich data that can help interpreting trial outcomes by adding details on a fuller spectrum of treatment results.

## Supplementary Information

**Additional file 1.**

## Data Availability

The datasets used and/or analysed during the current study are available from the corresponding author on reasonable request.

## Abbreviations

AR: Alba Realpe
FAI: Femoroacetabular impingement
HA: Hip arthroscopy
iHOT- 33: International Hip Outcome Tool
ISRCTN: International Standard Randomised Controlled Trials Number
NHS: National Health Service
NVIVO: Qualitative data analysis computer software
P#: Participant number
PHT: Personalised hip therapy
RCT: Randomised controlled trial
UK FASHIoN: UK Full randomised controlled trial of Arthroscopic Surgery for Hip Impingement versus best CoNventional Care
