# Supplementary material for: Patient experiences of receiving arthroscopic surgery or personalised hip therapy for femoroacetabular impingement in the context of the UK fashion study: a qualitative study
Source: Trials. 2021 Mar 16;22:211. doi: 10.1186/s13063-021-05151-6 (PMC7962311; doi:10.1186/s13063-021-05151-6)
Supplement: Supplementary file 1 — Additional file 1. [file 13063_2021_5151_MOESM1_ESM.docx]

**UK FASHIoN**

**Patient Interview**

**Chief Investigator: Professor Damian Griffin**

**Topic guide**

The aim of the interview is to explore patients’ experiences of care and the treatment they have received for hip impingement over the last 12 months. The in-depth interview will address the following questions:

1. What has it been like for you to have hip impingement?
2. How would you describe your current health status in relation to your hip?
3. Please describe the treatment you received for your hip impingement during the last 12 months.
4. What did you expect from the treatment you were allocated to?
5. Has the treatment fulfilled your expectations?
6. What are the advantages and disadvantages of the treatment you have received?
7. What impact has the treatment had on your life?
8. How do you feel about operative versus non-operative care after your experience?
9. Do you think you will require further treatment for your hip? Have you discussed further treatment with your healthcare provider?
10. In hindsight, which treatment would you have chosen? Why?
11. How was your experience participating in the FASHIoN study? Are there areas that could have been improved?
12. Would you recommend your friends and family to participate in FASHIoN or similar trials? Why?
